# Update on the pathogenesis of endometriosis-related infertility based on contemporary evidence

**DOI:** 10.3389/fendo.2025.1558271

**Published:** 2025-03-10

**Authors:** Qing Qi, Yaonan Li, Ziqin Chen, Zhihui Luo, Ting Zhou, Jing Zhou, Yanlin Zhang, Song Chen, Ling Wang

**Affiliations:** ^1^ School of Physical Education and National Equestrian Academy, Wuhan Business University, Wuhan, Hubei, China; ^2^ College of Chinese Medicine, Hubei University of Chinese Medicine, Wuhan, Hubei, China; ^3^ Yueyang Hospital of Integrated Traditional Chinese and Western Medicine, Shanghai University of Traditional Chinese Medicine, Shanghai, China; ^4^ Department of Obstetrics and Gynecology, Southern Medical University Nanfang Hospital, Guangzhou, Guangdong, China; ^5^ College of Acupuncture-Moxibustion and Orthopedics, Hubei University of Chinese Medicine, Wuhan, Hubei, China; ^6^ Department of Obstetrics and Reproductive Immunology, Shanghai East Hospital, Tongji University School of Medicine, Shanghai, China; ^7^ Department of Obstetrics, First Affiliated Hospital of Guizhou University of Traditional Chinese Medicine, Guiyang, China; ^8^ Center of Eugenics Research, First Affiliated Hospital of Guizhou University of Traditional Chinese Medicine, Guiyang, China

**Keywords:** endometriosis, infertility, endocrine, immune dysfunction, oxidative stress

## Abstract

Endometriosis, the most prevalent cause of infertility, is associated with anatomical distortion leading to adhesions and fibrosis, as well as endocrine abnormalities and immune disorders. This review discusses the mechanisms underlying endometriosis-related infertility. Firstly, alterations in the hypothalamic-pituitary-ovarian axis lead to the secretion of gonadotropins and steroid hormones, with adverse effects on ovulation and implantation, leading to fertility decline. Secondly, dysregulation of the hypothalamic-pituitary-adrenal axis induces elevated serum cortisol and prolactin levels in patients with endometriosis, accounting for its regulation of stress, depression, and anxiety. Abnormal interactions between endometrial cells and the immune system change the local microenvironment, resulting in epithelial-mesenchymal transition and inflammation. Activated epithelial cells, stromal cells, and immunocytes produce various chemokines, cytokines, or autoantibodies, creating an unfavorable environment for embryo implantation. These findings suggest that alterations in the immune spectrum play a crucial role in endometriosis-related infertility. Thirdly, oxidative stress has adverse effects on the ovarian reserve and subsequent embryonic development, predicting another promising strategy for endometriosis-related infertility. An unbalanced redox state, including impaired mitochondrial function, dysregulated lipid metabolism, and iron-induced oxidative stress, generates a pro-oxidative microenvironment, which negatively impacts oocyte quality and sperm and embryo viability. Thus, an updated understanding of the mechanisms involved in this disease will help to develop effective strategies to manage endometriosis-related infertility.

## 1 Introduction

Endometriosis is an estrogen-dependent disease characterized by the implantation of endometrial glands and extrauterine stroma, affecting 10% of women of reproductive age (1). Approximately 30–50% of women with endometriosis experience infertility, contributing to anxiety and depression in these patients and their partners (2). Mechanical damage caused by pelvic adhesions and ectopic ovarian cysts in women with advanced endometriosis can hinder oocyte release, obstruct the fallopian tubes, and impair the uterine environment for embryo implantation, resulting in female infertility (3). However, even in cases where there is no distortion of the pelvic anatomy, infertility is considered a possible consequence of early-stage endometriosis. The complex interactions among the endocrine, immune systems, and oxidative stress, collectively contribute to infertility.

There are still many questionable aspects concerning the pathogenesis of endometriosis-related infertility, making it a hot topic in current research. The pathological mechanism of subfertility in patients with endometriosis is complicated, including mechanical distortion caused by pelvic adhesion, reduced oocyte quality, gametal and embryonic toxicity, dysfunctional uterotubal motility, and altered endometrial receptivity. This review discusses the potential mechanisms underlying endometriosis-related infertility based on current evidence. This includes the involvement of the neuro-endocrine axis and sex hormones, the interaction between endometrial cells and the immune system, and oxidative stress. An updated understanding of the mechanisms involved in this condition will help to develop effective management strategies for endometriosis-related infertility.

## 2 The endocrine system

### 2.1 The hypothalamic-pituitary-ovarian (HPO) axis and related hormones

**Figure 1:**
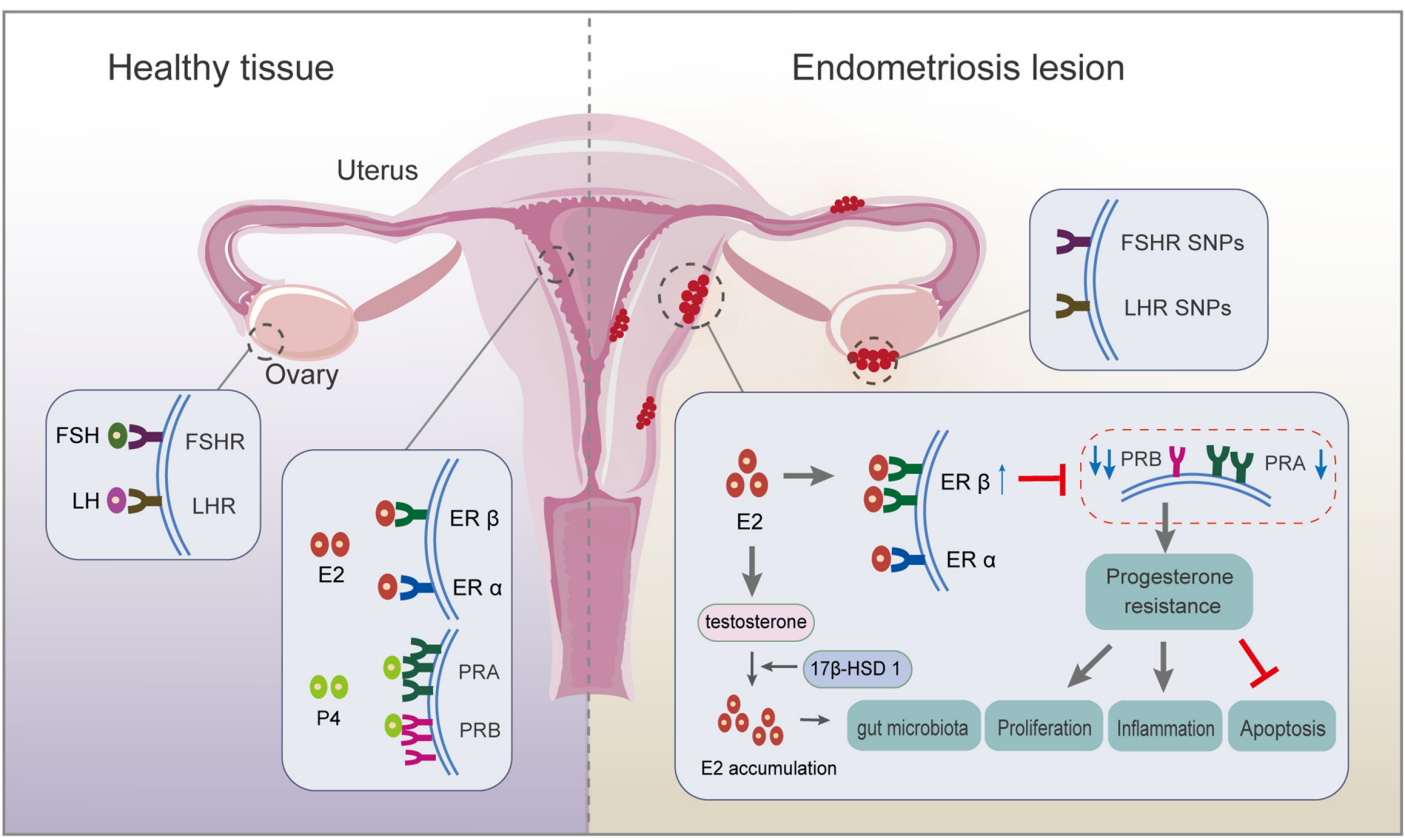
Respective roles of endocrine-related receptors in normal endometrial and endometriotic lesions. Firstly, SNP in the *FSHR* gene and *LHR* gene are associated with ovulation disorders. Deletion of *FSHR*, *LHR*, or both impedes granulosa cell survival and follicle development. Secondly, circulating E2 acts mainly on ERα; ERβ expression is upregulated, and ERα expression is weakened in endometriosis lesions. There is local accumulation of E2, mainly because of the ability of endometriotic lesions to synthesize E2, resulting from higher expression of 17β-hydroxysteroid dehydrogenase 1, a key enzyme for E2 production. Thirdly, PRA and PRB regulate the progesterone response. An imbalanced PRA: PRB ratio alters the progesterone pathway, inducing progesterone resistance, which finally inhibits apoptosis and promotes proliferation and inflammation. ER, estrogen receptor; FSH, follicle-stimulating hormone; FSH receptor, *FSHR*; LH, luteinizing hormone; LH receptor, *LHR*; PR, progesterone receptor; SNP, single nucleotide polymorphism.

Endometriosis is associated with alterations in the HPO axis (4), which tightly regulates the cyclic secretion of gonadotropins and steroid hormones, and the ovulation of the dominant follicle, preparing for subsequent fertilization and implantation. The development and maintenance of endometriosis are usually accompanied by abnormal fluctuations in these hormones and their receptors. For example, changes in gonadotropin ratios, estrogen dominance, and progesterone resistance caused by abnormal regulation of steroidogenesis are the main features of endometriosis-related infertility (5) (**Figure 1**).

#### 2.1.1 Follicle-stimulating hormone (FSH) and luteinizing hormone (LH)

The pituitary gland secretes FSH and LH, which travel via the bloodstream to the ovaries, where they synergistically induce folliculogenesis, oocyte maturation, ovulation, granulosa cell growth, and aromatase synthesis during reproduction (6). In patients with endometriosis combined with infertility, although the patients retain the ability to ovulate, there are significant disturbances in the rhythm and secretion of serum gonadotropic hormones, which severely affects their reproductive function. Evidence demonstrates reduced ovarian reserve function in endometriosis (7, 8): i) elevated serum concentrations of FSH and low concentrations of LH relative to FSH levels are observed in endometriosis, while lower FSH levels are potential indexes for successful postoperative pregnancy; and ii) single nucleotide polymorphisms (SNPs) in the FSH receptor (*FSHR*) gene and LH receptor (*LHR*) gene might be associated with ovulation disorders. The deletion of *FSHR*, *LHR*, or both, impedes granulosa cell survival and follicle development, ultimately altering oocyte quality through failure of cell maturation (4). A high incidence of mutations is observed in *LHR* in patients with endometriosis and infertility, suggesting impaired LH function in patients with endometriosis/infertility (9).

#### 2.1.2 Estrogen and estrogen receptors (ERs)

Endometriosis is an estrogen-dependent disease in which estrogen accumulation affects the progression of endometriotic lesions by binding to and activating ERs, including ERα and ERβ (10). Endometriotic tissue expresses aromatase and shows a high aromatase activity, partly accounting for the increased levels of E2 in endometrial tissue (11). Upregulated expression of 17β-hydroxysteroid dehydrogenase 1, a key enzyme for E2 production, is observed in the *in situ* endometrium, accompanied by high estrogen levels, which are responsible for a high rate of implantation failure in patients with infertile ovarian endometriosis (5). Overproduction of E2 drives the ERβ signaling pathway, leading to survival and inflammation of endometriotic tissue (11). Further study revealed that E2-induced estrogen in breast cancer 1, as an early prototypical estrogen-responsive gene, encodes a protein that modulates downstream gene expression to promote endometriosis development (12). The relative ratio of ERα to ERβ (ERα/ERβ) in endometriotic tissues is significantly lower than in the eutopic endometrium, accompanied by enhanced ERβ activity (13, 14). This results in evasion of endogenous immune surveillance for cell survival, inhibition of tumor necrosis factor (TNF)-α-induced apoptosis, an increase in interleukin (IL)-1β to enhance its cellular adhesion and proliferation properties, and strengthening of epithelial-mesenchymal transition (EMT) signaling (14). The close association of estrogen with inflammation is strongly supported by the estrogen-mediated expression of cytokines, such as IL-8, IL-6, IL-1β, and TNF-α, partly from macrophages (15). Thus, estrogen-macrophage interplay might be involved in endometriosis and infertility because of the remarkable ability of macrophages to respond to estrogens *in vitro*, probably creating a permissive environment for oocyte movement, fertilization, and implantation (16). Hence, estrogen pathways lead to an inflammatory state and disturbances in the immune environment, which affect morphological and biochemical changes associated with endometrial tolerance, resulting in pregnancy outcomes.

The interactions between high estrogen levels and the gut microbiota (GM) detrimentally affect embryo implantation and fertility by altering the cervical vaginal microenvironment, such as increased pH, bacterial overgrowth, and increased levels of inflammatory factors. The two major gut bacteria with significantly increased abundance, *Blautia* and *Dorea*, were shown to correlate positively with estrogen levels in patients with stage 3/4 endometriosis (17). The GM influences estrogen levels by regulating the enzymes responsible for estrogen metabolism (18). Estrogome, an estrogen-metabolizing enzyme, dissociates conjugated estrogen from bile acids by enhancing the activity of β-glucuronase, glucosidase, and hydroxysteroid dehydrogenase, resulting in increased levels of free estrogen in the peripheral blood (19). Uterine microbiota changes might affect the endometrium’s receptivity-related pathways during endometriosis (20). Metabolites produced by the GM might also modulate neurohormone release through the gut-brain axis. The neuroactive metabolites, such as short-chain fatty acids (21) and 5-hydroxytryptamine (22), resulting from GM metabolism can affect the brain’s neural signaling process through the circulatory system and influence downstream estrogen generation through the HPO axis (23). These metabolites activate gonadotropin-releasing hormone (GnRH)-producing neurons in the hypothalamus. The secreted GnRH signals to the pituitary gland increase the production of LH and FSH and stimulate the ovary to produce estrogen. Accordingly, when the intestine is in a biological imbalance, it is hypothesized to aggravate endometriosis-related infertility through the gut-brain axis (24). However, a causal relationship between microbiota and endometriosis cannot be established because of the insufficiency of current evidence and lack of further in-depth exploration.

#### 2.1.3 Progesterone and the progesterone receptor (PR)

Ovarian-derived progesterone regulates the periodic decidualization of endometrial stromal cells and facilitates the formation of an endometrial microenvironment that promotes blastocyst receptivity and embryo implantation. Excessive progesterone production during the follicular phase is involved in the abnormal signaling that impairs oocyte quality in endometriosis-associated infertility (25). Overexpression of progesterone is activated by beclin-1-induced autophagy in granulosa cells in patients with endometriosis (26). There are two functionally distinct isoforms of PR, PRA and PRB, which regulate the progesterone response. Regarding altered ER activity, undermethylation of the *ERB* promoter inhibits estrogen-induced PR expression, thereby reducing progesterone sensitivity (27). Abnormal overexpression of PRA and high DNA methylation level in the *PRB* promoter region in the endometriotic tissue of ovarian endometriosis patients increases the PRA: PRB ratio. Then, PRB, a strong transactivator in response to progesterone, loses its dominant position in the secretory nuclei, thus inducing progesterone resistance and leading to repeated implantation failure (5, 10). The imbalance of the PRA: PRB ratio alters the activity of progesterone and promotes the overexpression of specific response target genes, such as that encoding matrix metalloproteinase (MMP)-9, leading to extensive reconstruction of the endometrial layer and extracellular matrix, which directly affects the follicular microenvironment and the quality of oocytes and embryos. Mechanistically, dual inhibition of protein kinase B (AKT) and extracellular-signal-regulated kinase (ERK)1/2 pathways can reduce E2 biosynthesis and restore specific PRB signaling of endometrial cells (28). Moreover, how the interaction occurs between progesterone and E2 is still not fully understood. Further study demonstrated that mitogen inducible gene 6 mediates progesterone inhibition of E2 signaling by suppressing the Erb-B2 receptor tyrosine kinase 2 (ERBB2)-ERK signaling (29). However, the in-depth mechanism still requires further investigation.

### 2.2 The hypothalamic-pituitary-adrenal (HPA) axis and stress hormones

Dysfunction of the HPA axis has been identified in patients with endometriosis, being associated with the regulation of stress, depression, and anxiety (30). A significant correlation was found between cortisol levels and infertility and difficulties with sexual intercourse, with elevated serum levels of cortisol and prolactin being identified in patients with endometriosis-related infertility (31). This might explain why anxiety and depression are common in infertile patients and their partners (2). There are a number of possible explanations: i) neurons that secrete corticotropin-releasing hormone (CRH) are activated during stressful states, resulting in elevated plasma cortisol levels and prolactin release (30, 32); ii) neuroendocrine regulation and CRH release affected by chronic stress activate an inflammatory response, affecting the growth of an ectopic endometrium and the physiopathology of the pelvic organs, and leading to exacerbation of symptoms in endometriosis (33, 34); and iii) pleiotropy, such as SNP rs12666606 in *DGKB* (encoding diacylglycerol kinase beta), likely contributes to endometriosis-associated mental health (2).

## 3 Interaction between endometrial cells and the immune system

**Figure 2:**
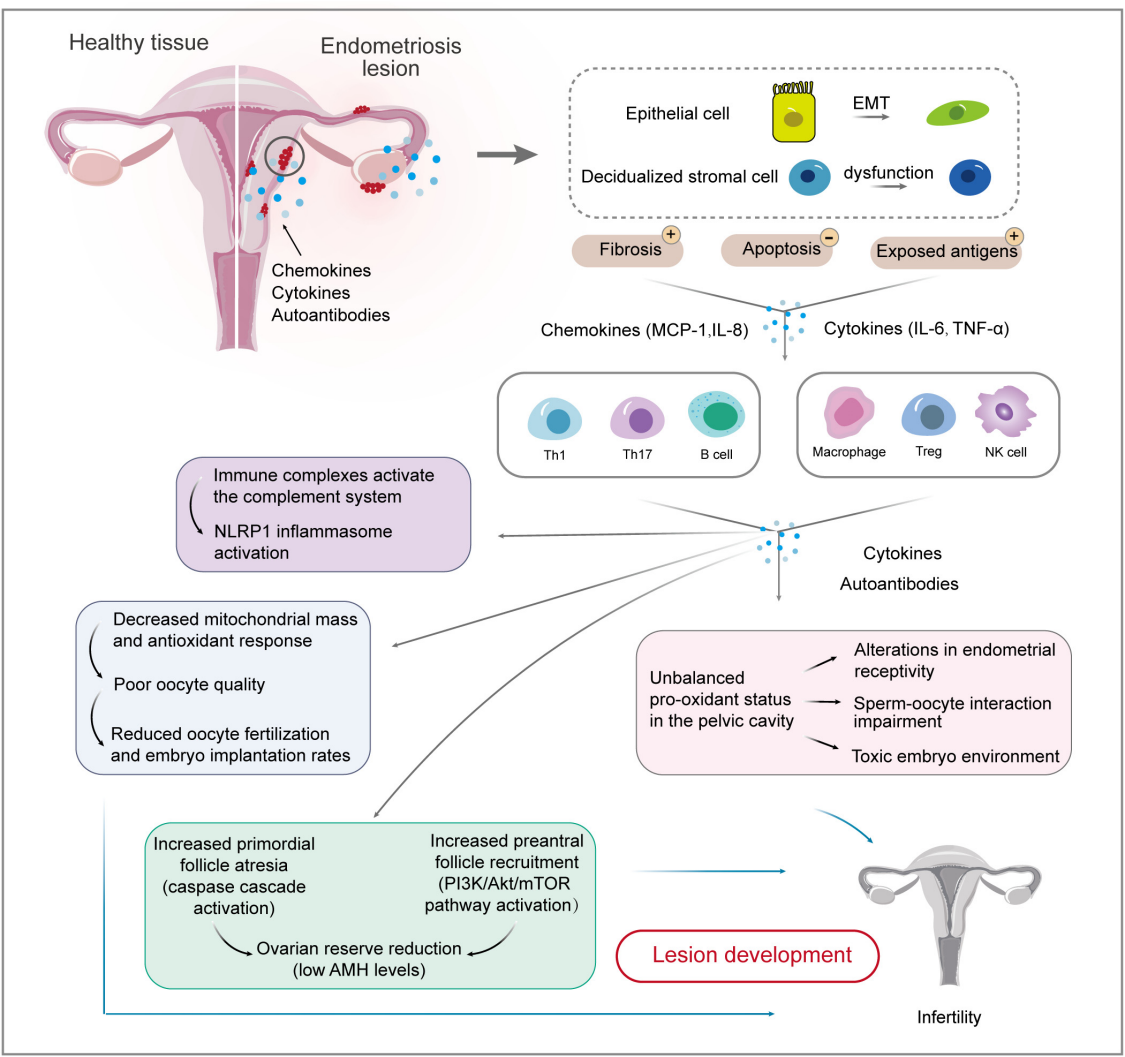
Interactions between endometrial cells and the immune system involved in endometriosis-related infertility. Firstly, causal relationships between endometrial cells and immune cells are well-established. EMT biological processes and stromal cell dysfunction account for fibrosis and exposed antigens, which cause ongoing inflammation and impaired immune surveillance. Immune cell populations, including T cells, macrophages, and NK cells, are involved in the development and maintenance of endometriotic lesions. The inflammatory environment of endometriosis induces immune complexes and an unbalanced pro-oxidant status, which affects ovarian reserve and oocyte quality, thereby reducing the chance of fertilization and implantation. EMT, epithelial-mesenchymal transition; IL, interleukin; MCP, monocyte chemoattractant protein; TNF, tumor necrosis factor.

A two-sample Mendelian randomization method identified specific immune cells involved in different types of endometrioses, uniquely establishing causal relationships between immune cells and endometriosis (35). High-resolution single-cell reference atlas profiled dysregulation in endometrial cells and inflammatory conditions in women with and without endometriosis (36, 37). Local or systemic inflammatory response dominates the pathological progression of endometriosis. It compromises the reproductive capacity by affecting folliculogenesis and oocyte maturation (38), suggesting the critical role of immune profile alteration in endometriosis-related infertility (**Figure 2**).

### 3.1 Endometrial cells

#### 3.1.1 Epithelial cells and EMT

The pathophysiology of endometriosis involves EMT, a biological process in which polarized epithelial cells transform to highly motile mesenchymal cells (39). Globally, plastic consumption has been rapidly increasing, and persistent pollutant exposure, such as plasticizers, has increased endometriosis-related EMT development, contributing to disease onset and progression (40). Mesothelial-to-mesenchymal transition (MMT), a type II EMT process, occurs in mesothelial cells that lose their polarity and take on a fibroblast-like phenotype in a pathological state (41). It was observed that MMT can trigger peritoneal fibrosis, angiogenesis, and implantation of an ectopic endometrium, in which peritoneal mesothelial cells undergoing MMT can lead to cell adhesions (41). Mechanistically, EMT production is associated with the pathophysiology of fibrosis through the IL-33-suppression of tumorigenicity 2 (ST2) axis, the transforming growth factor (TGF)-β signaling pathway, and the phosphatidylinositol-4,5-bisphosphate 3-kinase (PI3K)/AKT/glycogen synthase kinase 3 beta (GSK-3β)/β-catenin axis, which promote the migration and invasion of endometrial mesenchymal cells and mediate peritoneal adhesions (39–42). Targeting the IL-33-ST2 axis might be a feasible method to control fibrosis in endometriosis (39). Epithelial cells enhance ectopic endometrial cell survival and apoptotic resistance, decrease natural killer (NK) cell activity, and induce macrophage recruitment by overexpressing TGF-β1. This promotes fibrosis and adhesion formation by upregulating tissue inhibitor of metalloproteinase 1 expression and inducing myofibroblast differentiation (43). TGF-β-dependent signaling, activated by soluble endoglin and growth differentiation factor 15, promotes inflammatory chemokines in the ovary and pelvic adhesions, contributing to reduced fertility (44). In conclusion, the EMT theory provides a rationale for the involvement of endometrial cell transformation in the development of endometriosis-associated infertility.

#### 3.1.2 Stromal cells and pro-inflammatory factors

Decidualized stromal cells are highlighted in endometriosis (36). Endometrial cells account for the increased number of peritoneal macrophages by producing monocyte chemoattractant protein (MCP)-1 and IL-6 (45). MCP-1 has a critical function in the pathogenesis of endometriosis-associated infertility, especially in the early stages, being implicated in the regulation of follicular growth, ovulation, luteal development, and the induction of intrafollicular inflammatory state, as well as being a potential predictor of worse prognosis (46). However, distinguishable changes in MCP-1 levels were also detected between patients with endometriosis and those without, which was independent of cycle and fertility status (47).

In addition, endometrial stromal cells account for IL-6 and IL-8 production in endometriotic tissue (48), affecting blastocyst adhesion and inhibiting embryo outgrowth to a great extent (49). High levels of IL-6 are detected in follicular fluid from naturally mature follicles in patients with moderate and severe endometriosis, which is associated with lower pregnancy rates (50). The reasons may be that, i) high levels of IL-6 might affect follicular biology and reduce oocyte quality (50); and ii) the combination of IL-6 and its soluble receptor significantly reduces sperm motility (51). Therefore, it is hypothesized that combining IL-6 and IL-8 with serum carcinogenic antigen-125 levels, a major biomarker of endometriosis, could predict infertility in patients with endometriosis (52). Moreover, IL-8 blockade is hypothesized to reduce fibrosis in endometriosis (53). The data related to these biomarkers need to be validated in future studies, followed by testing of their clinical utility for routine screening, as non-surgical diagnostic markers, and as targets for immunomodulatory therapy, with the aim of identifying immunomodulatory strategies for this disease.

Endometrial stromal cells serve as progenitors of adenomyosis, in which multiple WNT proteins originating from fibroblasts, epithelial cells, and endothelial cells constitute a critical paracrine network (54). The hippo/yes-associated protein 1 (YAP1) signaling pathway plays an important role in endometriosis. YAP1 reduces PR expression and inhibits endometrial stromal cell decidualization by upregulating the expression of the microRNA miR-21-5p (54). In addition, methyltransferase-like 3-regulated m6A modification impairs the decidualization of endometrial stromal cells by regulating degradation of the mRNA encoding Forkhead box O1, a core decidualization marker in endometrial stromal cells (55, 56). Activation of the AKT/mechanistic target of rapamycin kinase (mTOR) pathway promotes endometrial stromal cells to secret stromal cell-derived factor-1 (SDF-1), which enhances the migration and recruitment of endothelial progenitor cells in the SDF-1-C-X-C motif chemokine receptor 4 (CXCR4) axis (57). Moreover, endometrial fibroblasts exhibit pro-inflammatory characteristics, with activated IL-8, cellular communication network factor 2, and transgelin expression, which organize cell migration and the extracellular matrix (58). The coculture with endometrial stromal cells induced depressed cytotoxicity and aberrant metabolism of CD8+ T cells via signal transducer and activator of transcription (STAT)1/programmed cell death (PDCD)1 pathway; correspondingly, CD8+ T cells depressed the proliferation of ectopic stromal cells (ESCs) through inhibiting cyclin-dependent kinase (CDK)1/cyclin B (CCNB)1 pathway to arrest the cell cycle and triggered inflammation (59). Hence, in endometriosis, pro-inflammatory pathways are dysregulated in epithelial and stromal cells (60).

### 3.2 Immunocytes

The presence of ectopic endometrial implants may cause ongoing inflammation and impaired immune surveillance, which might be responsible for maintaining the dysregulated state and impaired reproductive function (53). Immunocytes account for cytokine activation in developing endometriosis-related infertility (47). Aberrant distribution of immune cell populations, including macrophages, NK cells, T cells, and B cells, is often observed in the peritoneal fluid and eutopic endometrium of patients with endometriosis, resulting in chronic inflammation and an inhospitable environment for embryo implantation, thus contributing to endometriosis-associated infertility (61).

#### 3.2.1 Macrophages

An integrated single-cell reference atlas highlighted the critical function of macrophages in endometriosis (36). The pro-inflammatory M1 phenotype and a propensity for M2 to M1 polarization were observed in the endometrium of patients with endometriosis (62). However, as the disease progresses, macrophages polarize toward the M2 phenotype (63), evidenced by M2 macrophage accumulation in adenomyosis, which enhances the invasion capacity of adenomyotic cells (64). Moreover, M0 to M2 transition caused by gut dysbiosis-derived β-glucuronidase promoted the proliferation and migration of endometrial stromal cells (65). In accordance with M2 polarization, the inverse correlation between aggravating indicators of endometriosis and the expression of pattern recognition receptors, toll-like receptor 4 (TLR4), and receptor of advanced glycation end-products on macrophages indicates the presence of immunosuppression in endometriosis (66). Subsequently, downstream events are activated. Vascular endothelial growth factor (VEGF)-A release and blood vessel formation both increase, facilitating Schwann cell migration and peripheral axon regeneration (67). Interestingly, repolarization of M2 to M1 can inhibit ectopic endometrial cell growth (68).

Infertile patients with endometriosis showed altered expression of macrophages in the endometrium and increased numbers of uterine NK (uNK) cells or plasma cells at higher concentrations of macrophages (69). Activation of insulin like growth factor binding protein 5 (IGFBP5)+ macrophages with pro-inflammatory properties is notably observed in ovarian endometrioma (58). Increased serum levels of TNF-α, IL-6, and IL-1β, released from disease-associated macrophages, were observed in patients with endometriosis-induced infertility (70, 71), creating a feedforward loop to aggravate endothelial cell activation. IL-1β inhibits ERα and PRs in primary human endometrial stromal cells via activation of the ERK1/2 pathway, ultimately inhibiting decidualization (72). Administration of an anti-IL-1 receptor 1 (IL-1R1) antibody or IL-1R1-associated kinase 4 inhibitor limited endometrial lesions or suppressed lesion formation (73). Anakinra, an IL-1 inhibitor and an FDA-approved injectable medication to treat rheumatoid arthritis, was proposed to reduce pelvic pain caused by endometriosis in a human pilot study; however, no results were posted after the study (74). TNF-α facilitates endometrial cell proliferation in ectopic environments (75). Peri-implantation treatment with a TNF-α inhibitor increased the implantation and clinical pregnancy rates, while the ongoing pregnancy and live birth rates were not significantly different (76). However, the safety of TNF-α inhibitors on embryos and fetuses has not been fully clarified.

In addition, macrophages regulate ectopic lesion growth and angiogenesis in an autocrine and paracrine manner via extracellular vesicles (77). The endothelial cell-macrophage interaction facilitates epithelial cell proliferation and results in sterile endometrial inflammation (78). Hence, therapeutic strategies, such as suppression of ‘inflammation,’ might dysregulate the cross-regulation of ‘pro- and anti-inflammatory mediators,’ leading to detrimental effects in patients with endometriosis (43).

#### 3.2.2 uNK cells

In the uterus, resident uNK cells play a crucial role in successful pregnancy by generating and secreting angiogenic factors, such as VEGF and angiotensin 2, and promoting revascularization, vasodilation, and neovascularization in the maternal endometrium and trophoblast cell invasion into the endometrium. Thus, defects in uNK cell activity or dysfunction of uNK cells might affect embryo implantation and development, resulting in decreased fertility and adverse pregnancy outcomes. NK cell exhaustion with aberrant upregulation of apoptosis-related genes has been identified in ovarian endometrioma (58). However, the increased number of CD16+ cytotoxic uNK cells observed in the eutopic endometrium of patients with endometriosis exhibit higher toxic activity and produce cytotoxic factors in response to trophoblast cells, possibly leading to infertility (79). Similarly, a concomitant increase of endometrial CD56+ NK cells was found in patients with endometriosis (58). Based on these studies, an altered proportion of uNK cells might also be responsible for an excessive inflammatory and hypoxic environment during embryo implantation or decidualization, thereby increasing the risk of infertility; however, more studies are needed to understand the exact mechanisms by which uNK cells affect fertility.

#### 3.2.3 T cells

Elevated levels of IL-17A, an inflammatory cytokine produced by T helper (Th)17 cells, are found in serum and follicular fluid to varying degrees when endometriosis and infertility co-exist (80). As a stimulator of recruitment and activation of immune cells, such as monocytes and neutrophils, upregulation of IL-17 could further activate many downstream cytokines, such as IL-1, IL-6, IL-8, and MCP-1, and participate in the occurrence of endometriosis-related infertility by aggravating inflammation, enhancing toxic effects, and affecting the function of the female reproductive system. Peritoneal fluid-derived IL-17 induces monocytes to differentiate into M2 macrophages (81), contributing to an immunosuppressive environment in the pelvic cavity and promoting the proliferation, growth, invasion, and immune escape of ectopic lesions. The involvement of IL-17A and Th17 cells in the preservation of endometrial cell viability is achieved through the suppression of apoptosis and the inhibition of NK cell-induced cytotoxicity toward endometrial cells, mediated by the ERK1/2 pathway (82). Extracellular vesicles from individuals with endometriosis-associated infertility significantly inhibited the total motility, progressive motility, linear velocity, and the acrosome reaction rate of human sperm by inducing the production of inflammatory cytokines leading to a Th17/regulatory T cell (Treg) imbalance (83).

Dysregulation of T lymphocyte homeostasis, especially deficiency in the number and activity of Tregs, might be one of the causes of endometriosis predisposing to the development of infertility (84). Tregs might induce type 2 immunity in the endometriotic microenvironment, likely suppressing the recruitment of immune cells, which can recognize and target endometrial antigens, contributing to the progression and fibrogenesis of endometriosis (85). Consistently, Th2-skewing occurs in endometriotic lesions, whereas Th1-skewing occurs in peripheral blood and peritoneal fluid (86). Treg dysfunction might exaggerate local inflammation and stimulate angiogenesis by interacting with effector T cells and macrophages, thereby facilitating the attachment and growth of endometrial implants and increasing the risk of reproductive and perinatal complications.

#### 3.2.4 B cells

Studies have shown an association between endometriosis and autoimmune diseases (87). Various autoantibodies, including anti-histone H1.2 and anti-alpha 2-HS glycoprotein, were found in the peritoneal fluid or serum in infertile patients with endometriosis, affecting 60% of women with endometriosis-associated infertility (88, 89). Women with recurrent miscarriages have higher levels of anti-laminin-1 antibodies compared with those in healthy controls in the early stages of pregnancy (90). Anti-β2-Glycoprotein I/HLA-DR antibodies might be associated with the pathophysiology of infertility and endometriosis (91). In addition, immune complexes activate the complement system and induce NLR family pyrin domain containing 1 (NLRP1) inflammasome activation to release IL-1β, thus dysregulating pro-inflammatory pathways (60, 92). Complement C1q is the first recognition molecule of the complement classical pathway. High expression of C1q in endometriotic lesions induces a robust proangiogenic effect on endothelial cells that overexpress the receptor for the globular heads of C1q (93). The mechanisms involve linker for activation of T cells family member 2 (LAT2, also known as NTAL and LAB)-mediated calcium mobilization, TGF-β, integrin-mediated cell adhesion, platelet-derived growth factor, and Rac family small GTPase 1 (RAC1)/P21 (RAC1) activated kinase 1 (PAK1)/p38/MMP2 signaling (89). Enriching the LAT2 pathway in activated B cells and monocytes supports the involvement of B cell-mediated autoimmunity in endometriosis-associated infertility (89). It is likely that Th2 polarization at ectopic sites and the consequent generation of IL-4 are also triggering factors for the appearance of these autoantibodies (94). In light of these findings, serum IgA and IgG anti-endometrial antibody levels might predict the pregnancy outcome for patients with endometriosis and tubal factor infertility (95).

Pyroptosis, a novel form of inflammatory cell death, was revealed to have a significant but neglected role in endometriosis via bioinformatic analysis of published data from human and animal models. Pyroptosis exacerbates immune dysfunction by recruiting activated immune cells, including macrophages, neutrophils, central memory CD8+ T cells, and Tregs with unregulated chemokines (96). It is worth noting that the complex inflammatory milieu results from the synergistic activity among different immune cell populations rather than a single role. The question of whether immune dysfunction serves as a pathogenetic mechanism leading to endometriosis-associated infertility or is a consequence of the disease remains unknown.

Various immune cells and the inflammatory cascade are involved in the development and maintenance of endometriosis; therefore, more work is required to determine the mechanisms of endometriosis-related signaling pathways. Several endometriosis-related signaling pathways have been discovered, including nuclear factor kappa B (NF-κB), mitogen-activated protein kinase (MAPK), fibroblast growth factor receptor (FGFR), PI3K/AKT/mTOR, YAP, Wnt/β‐catenin, Rho/Rho associated coiled-coil containing protein kinase (ROCK), and TGF‐β (97). NF-κB plays a crucial role in the immune response as a node in the main inflammation-activated signaling pathway, which facilitates signaling between IL-1 and TNF-α receptors and the production of leukemia inhibitory factor and IL-6 (98). Inhibition of NF-κB signaling reduced endometrial stromal cell proliferation, migration, invasion, EMT, and inflammation in endometriosis (99). The MAPK signaling pathway regulates cell proliferation and invasion, and the Ras-related protein Rap-1 (RAP1) pathway interacts with the MAPK pathway to promote endometriosis development (97). FGFR2 promotes the proliferation, migration, and invasion of ESCs via activation of the ERK signaling pathway in endometriosis: an ERK1/2 inhibitor could counteract the effects of FGFR2 on ESC proliferation and invasion (100). Signaling pathways have been confirmed to be involved in endometriosis; therefore, new strategies should be developed to target these pathways.

## 4 Oxidative stress

Chronic low-grade inflammation can induce oxidative stress. Reactive oxygen species (ROS) derived from cytokines and immunocytes in the follicular fluid microenvironment play a dual role in female reproduction. Altered oxidative stress was observed in patients with endometriosis (101). In addition, differentially expressed genes related to oxidative phosphorylation and mitochondrial function were highlighted for oocyte competence acquisition in cumulus cells of endometriosis (102). Oxidative stress, resulting from the duration and severity of exposure to pro-oxidant molecules and antioxidant defenses, can damage the ovarian reserve and adversely affect oocyte and subsequent embryo development. Elevated systemic oxidative stress can lead to notable fibrinogen oxidation and structural alterations, which are associated with impaired fibrin polymerization and enhanced resistance to plasmin-induced lysis. These changes might increase the thrombotic risk in women with endometriosis, which is detrimental to pregnancy (103).

### 4.1 Oxidative stress and mitochondrial function

Oxidative stress plays a key role in the pathogenesis of endometriosis-related infertility. High levels of oxidative stress-sensitive markers, such as advanced oxidation protein products and myeloperoxidase, found in endometriosis patients’ follicular fluid, severely impair mitochondrial metabolism and spindle formation during oocyte meiosis, thereby accelerating follicular depletion, reducing oocyte quality, and lowering fertilization rates (104, 105). High-quality oocytes, the prerequisite for successful pregnancy, are closely related to mitochondrial function and an enzymatic environment that balances intracellular ROS levels (106). However, abnormal mitochondrial accumulation, such as swelling and cristae disruption, is observed in oocytes from women with endometriosis, which would significantly affect energy supply and lead to oocyte stagnation and degeneration during fertilization (107). One explanation might be the elevated expression of prohibitin 1, a highly conserved protein related to mitochondrial function, which leads to increased glucose consumption and lactic acid production, as well as aberrant expression of glycolysis-related enzymes, in the granulosa cells of patients with endometriosis (108). In contrast, ectopic endometrial stromal cells maintain high-energy mitochondrial metabolism through the antioxidant effect of superoxide dismutase overexpression, and promote proliferation and migration in patients with ovarian endometriosis (107). The local microenvironment comprises key constituents to which gametes and preimplantation embryos are exposed (109). High ROS activities and associated oxidative stress in the follicular fluid and serum from patients with endometriosis have the ability to damage gametes and embryos (109). Ovarian endometrioma, one of the major subtypes of endometriosis, leads to a loss of the ovarian cortex-specific stroma and decreased follicle density through occupying compression and local inflammatory invasion, which was confirmed by the presence of early follicular atresia and increased expression of apoptosis-related proteins in ovarian cortical biopsies (110).

### 4.2 Oxidative stress and dysregulated lipid metabolism

A multi-omics study using a mouse model and women with endometriosis revealed the dysregulation of lipid metabolism, identifying 55 upregulated and 67 downregulated metabolites (111). The mechanism involves cellular reactive oxidative stress, cell proliferation inhibition, senescence, apoptosis, and regulation of MAPK-ERK1/2 signaling, leading to a reduced number of mature oocytes (111). Upregulated expression of senescence-associated β-galactosidase can aggravate endoplasmic reticulum stress and differentially affect senescence-associated secretory phenotype factors (IL-1β, MMP-9, and keratinocyte growth factor), which are useful indexes to evaluate the oocyte retrieval number and mature oocyte number. The changes further promote the senescence of ovarian granulosa cells (112). Sphingosine 1-phosphate (S1P) is a metabolite of sphingolipid and an inflammatory mediator. The S1P concentration significantly increased during the non-menstrual phase in patients with endometriosis compared with that in the controls. Further experiments showed that S1P administration increased the size of the endometriotic-like lesion via biasing peritoneal macrophages toward M2 polarization and increasing the expression of IL-6 and mitochondrially encoded cytochrome C oxidase II (113).

Lipid rafts, an acceptor of TLR4, are micro-structure areas on the membrane that are rich in cholesterol. Lipid raft levels increased markedly in uterine tissues after lipopolysaccharide (LPS) treatment, accompanied by the production of inflammatory factors (114). Activation of the liver X receptor-alpha (LxRα)-ATP binding cassette subfamily A member 1 (ABCA1) pathway might account for the outflow of cholesterol (114). Moreover, lipophagy, a special form of autophagy, can deliver the lipid cargo within the lipid droplets to lysosomes for degradation to release free cholesterol (115). In addition, density lipoprotein receptor (*LDLR*) genes are overexpressed in the foci of deep bowel endometriosis, according to an exploratory case-control study (116), supporting the feasibility of LDLR-targeted therapy in deep endometriosis. Meanwhile, an apolipoprotein E mimetic decreased the lesion size and modified the macrophage phenotype in gain of function experiments (117). Lipid-soluble statins, exemplified by simvastatin, lovastatin, and atorvastatin, effectively inhibit the growth and invasiveness of primary human endometrial stromal cells compared with that of pravastatin, a water-soluble statin (118). Five mg/kg atorvastatin has been shown to regress the ectopic lesions without discernible harm to the ovary in endometriosis mice (119). However, further study is needed to verify the preclinical results.

### 4.3 Oxidative stress and iron toxicity

Extremely high levels of iron are found in peritoneal fluid, ectopic cyst fluid, and ovarian follicles of patients with endometriosis because of numerous lysed erythrocytes in accumulated refluxed menstrual blood (120). In the presence of transferrin insufficiency and iron overload, iron toxicity induces oxidative stress through the Fenton reaction, the most biologically potent ROS reaction, to produce superoxide anions and hydroxyl radicals, resulting in direct damage to follicles, oocytes, sperm, and embryos (121, 122). The iron overload-induced oocyte damage and embryo toxicity might be related to ferroptosis mediated by multiple signaling pathways, including nuclear receptor coactivator 4-dependent ferritinophagy (123), glutathione peroxidase 4-dependent downregulation (124), and heme oxygenase 1 (HMOX1) overexpression (120), in which mitochondrial dysfunction and structural damage play a central role. HMOX1 regulates the delicate balance of iron-induced oxidative stress in endometriotic cyst fluid (125). However, when ROS production exceeds the capacity of cellular antioxidant defenses to remove these toxic agents, oxidative stress occurs, as confirmed by elevated specific biomarkers of oxidative damage in cyst fluid, including lactose dehydrogenase, lipid peroxidation, and 8-hydroxy-2’-deoxyguanosine (126). This explains why the severity of endometriosis-related infertility can be predicted to some extent by measuring iron levels in ectopic cysts. Moreover, iron overload-induced gasdermin E (GSDME)-mediated pyroptosis can trigger the activation and release of IL-16, which drives inflammation in ovarian endometriosis through CD4^+^ T cells. Indeed, compound Z30702029 inhibited GSDME-N-terminal domain-mediated pyroptosis in preclinical experiments (127).

As mentioned above, it is reasonable to conclude that a pro-oxidant microenvironment in the local vicinity of the follicle adversely affects the quality of oocytes, sperm, and embryos. The mobilization of potent antioxidants might protect them from oxidative damage. Coenzyme Q10 improved the *in vitro* maturation of oocytes exposed to follicular fluid from patients with endometriosis (128). The administration of vitamins E and C significantly decreased the myeloperoxidase levels in follicular fluid, but not plasma, in moderate/severe endometriosis (105). Rapamycin, used as an anti-senescence drug, could elevate *in vitro* fertilization (IVF) outcomes, including embryo transfer success and fertility rates via IVF (129). Thus, antioxidant stress might be a promising strategy to target oxidative stress to inhibit endometriotic lesion progression and alleviate endometriosis-associated symptoms, including infertility.

## 5 Conclusion

Although the onset of endometriosis-associated symptoms often occurs during adolescence and adulthood, the disease occurs across the life course. Early assessment of risk factors associated with endometriosis could help clinicians to make a diagnosis and reduce diagnostic delay.

An updated understanding of the pathogenesis of endometriosis is needed to develop medical approaches. In recent years, researchers have progressively elucidated the mechanisms underlying endometriosis-related infertility, summarizing multiple factors that influence patient fertility. Firstly, estrogen dominance and progesterone resistance, along with abnormal steroidogenesis, are hallmark features of endometriosis-associated infertility. Additionally, dysregulation of the HPA axis contributes to elevated serum cortisol and prolactin levels in patients with endometriosis. Secondly, ectopic lesions facilitate chronic inflammation, creating an unfavorable environment that is associated with the release of various inflammatory factors. The latter stimulates angiogenesis and induces the further spread of the lesions. Furthermore, interactions among endocrine functions and immune responses play a crucial role. Therefore, maintaining the balance among these systems is essential for optimal endometrial function. The GM, as a central regulator of inflammatory and proliferative conditions, is involved crucially in the onset and progression of endometriosis. The disease involves a multi-system disorder and hormone dysregulation, which might open the door for inter-organ crosstalk mediated by the GM-endocrine system for endometriosis (130, 131). A genetic correlation between endometriosis and gastrointestinal disorders was reported for the co-existence of endometriosis and gastrointestinal symptoms. Based on the shared pathogenesis, including estrogen regulation and inflammation, potential therapeutic drug targets (cholecystokinin B receptor and phosphodiesterase 4B) were identified, providing evidence of shared disease etiology and implicating clinical diagnostic and treatment decisions for both diseases (131). More studies are required to further explore the connection between endometriosis and gastrointestinal disorders and the novel view of the pathogenesis of endometriosis. Thirdly, an unbalanced redox state generates a pro-oxidative microenvironment around follicles, negatively impacting oocyte quality, and sperm and embryo viability. Iron-induced oxidative stress and ferroptosis within granulosa cells lead to suboptimal oocyte development—a focus area for current research endeavors.

Endometriosis is a heterogeneous disease with a complex pathogenesis; therefore, a single-molecular model is insufficient to explain endometriosis-related infertility. A complex *ex vivo* endometrial model was developed to recapitulate the organization and properties of the human endometrium using innovative tissue bioengineering methods (132). This preclinical approach is hypothesized to predict individual responses to different types of hormonal treatment, providing a better understanding of their effects. Furthermore, much work has been done to develop biomarkers in blood and endometrial tissue, such as microRNAs, B-cell lymphoma 6 protein, and sirtuin 1 protein immunostaining (133). However, most of the non-surgical diagnostic techniques remain to be developed or at least fully validated.

With improving insights into endometriosis pathophysiology, further advances in treatment strategies will emerge. Can diet or supplements have an impact on endometriosis-associated symptoms? Studies have reported generally positive responses to a range of dietary modifications. Changes in diet might affect symptoms through anti-inflammatory effects and a contribution to a more favorable GM. Selenium intake alleviates LPS-induced endometritis by reducing cholesterol levels (114). Copper’s chelation with ammonium tetrathiomolybdate showed a highly effective antiproliferative and antiangiogenic effect by modulating the oxidative imbalance in a murine model of endometriosis. The results indicate an anti-endometriotic potential as a possible non-hormonal treatment (134). However, most studies were conducted for a short time on small groups of individuals, often without a control group (135). Novel delivery systems, such as mifepristone-loaded nanostructured lipid carriers (NLCs), have been developed as an effective strategy for endometriosis-targeted therapy (136, 137). Furthermore, NLC will benefit certain drugs that are difficult to dissolve (137). A targeted nano delivery system as the carrier of mifepristone could selectively target M2 macrophages highly enriched in ectopic endometrial tissue via the secreted protein acidic and cysteine rich receptor. The targeting strategy reduces side effects by increasing the local drug concentration (68).

Endometriosis is associated with a reduced quality of life, and its heterogeneous presentation impedes diagnosis and treatment. Moreover, current medical management, including surgical and medical methods, is ineffective for a sizable proportion of women (138). Women often endure symptoms without good health treatments because of their complex pathogenesis. Global public health policies are urgently needed to promote awareness and implement multidisciplinary care for patients with endometriosis (139). In particular, self-management strategies should be investigated to improve patients’ overall quality of life.
